# CAIPIRINHA-accelerated 2D bSSFP imaging with improved banding behavior using Gradient-Controlled Local Larmor Frequency (GC-LOLA)

**DOI:** 10.1186/1532-429X-18-S1-P301

**Published:** 2016-01-27

**Authors:** Peter Speier, Daniel Staeb, Edgar Mueller

**Affiliations:** 1 Siemens Healthcare GmbH, Erlangen, Germany; 2 grid.8379.50000000119588658Department of Diagnostic and Interventional Radiology, University of Wuerzburg, Wuerzburg, Germany; 3 grid.1003.20000000093207537Centre for Advanced Imaging, The University of Queensland, Brisbane, QLD Australia

## Background

Cardiac MRI often requires a careful trade-off between SNR, spatio-temporal resolution and slice coverage. Providing fast acquisitions, high SNR and flow/motion robustness, bSSFP has become the dominant sequence. Drawbacks are high SAR levels and sensitivity to B0 inhomogeneities. For improving slice coverage, MS-CAIPIRINHA [1] has emerged as a standard method. By simultaneously scanning multiple slices, it provides acceleration in slice direction with minimal SNR penalty.

When combining MS-CAIPIRINHA with bSSFP, the sequence steady-state and contrast have to be maintained. Two methods are available: The first [2] employs RF-based multi-slice encoding [1], generating slice-specific shifts in the bSSFP pass-band structure effectively reducing the off-resonance robustness by a factor of two. The second [3] applies balanced gradient encoding during readout, potentially increasing the sensitivity to eddy currents for small inter-slice distances because encoding changes from excitation to excitation.

## Methods

**Figure 1:**
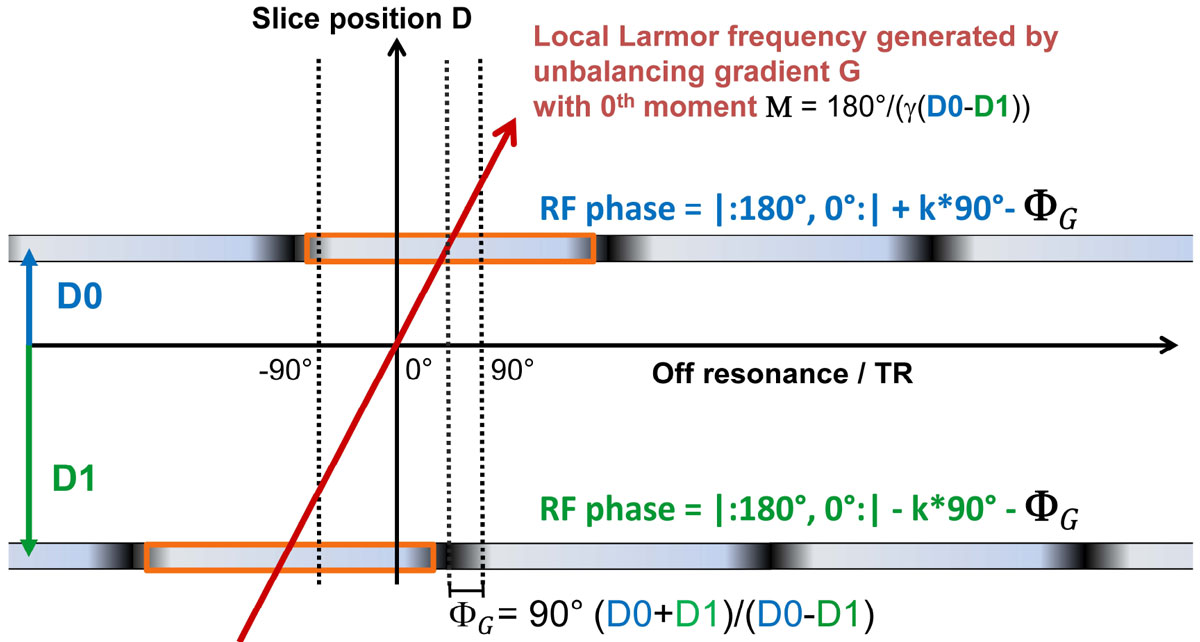
**bSSFP with GC-LOLA for two slices**.

We propose a new method, called gradient-controlled local larmor adjustment (GC-LOLA), that eliminates the drawback of the RF-encoded combination in two steps: (1) By slightly unbalancing the slice select gradient, the Larmor frequency is made locally dependent, which compensates the relative shift between the pass-bands. (2) In addition, the RF phase cycles are modified to shift the centers of the aligned pass-bands to resonance. The method is illustrated in Figure 1 for two slices S0 and S1 at slice positions D0 and D1. The pass-band shifts are fully corrected by unbalancing the slice gradient by the moment **M**, distributed evenly on slice pre- and rephaser, and subtracting the residual off-resonance **Φ**_G_ from the RF phase increments in both slices. To test the concept, phantom and in-vivo measurements were performed using a bSSFP sequence prototype, modified in-house to support MS-CAIPIRINHA and GC-LOLA (MAGNETOM Aera and Skyra, Siemens Healthcare GmbH, Erlangen).

## Results

**Figure 2:**
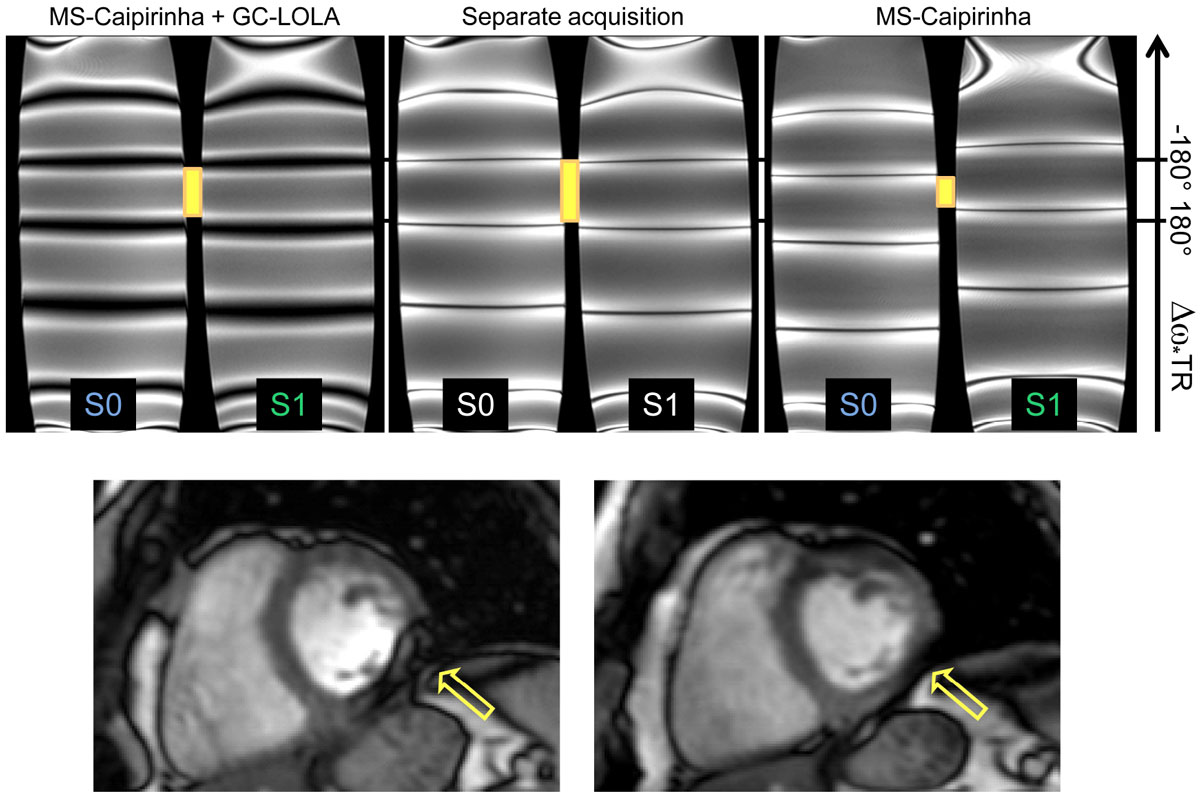
**Top row: bSSFP band structure in a phantom with linear gradient for RF-encoded MS-CAIPIRINHA with two simultaneously excited slices without (left) and with GC-LOLA (right) compared to two single-slice acquisitions (center)**. Conventional MS-CAIPIRINHA shifts the stop-bands by +¼ and -¼ of the band distance in slice 1 and 2, respectively. The application of GC-LOLA shifts the stop-bands back to their original positions and blurs them. The common central pass-band is indicated with orange bars. **Bottom row:** One of two slices of an MS-CAIPIRINHA scan of a healthy volunteer at 3T without (left) and with (right) GC-LOLA. The stop-band indicated by the arrow has successfully been moved out of the posterior wall.

The Phantom results (Figure 2 top row, slice thickness 5 mm, slice positions S0: 55 mm, S1: 70 mm, flip 40°) demonstrate the successful restoration of the original band pattern. Due to the gradient unbalancing, the stop-bands appear slightly blurred. The benefit of increasing the off-resonance robustness can be seen from the volunteer scan (Figure 2 bottom row, slice thickness 5 mm, gap 100%, TR 2.9ms, TE 1.3ms): A stop-band is shifted out of the posterior of the left ventricle (LV) and the signal in the LV blood pool is more homogeneous.

## Conclusions

Our preliminary results indicate that GC-LOLA stabilizes MS-CAIPIRINHA-accelerated bSSFP with respect to field inhomogeneities, without the need for toggled gradients from TR to TR.
